# Miscellanies

**Published:** 1844-10-01

**Authors:** 


### Miscellanies.

### RELATIVE FREQUENCY OF PULMONARY PHTHISIS.

DR. BOYD has published the following curious statistical report from the St. Marylebone Infirmary.

"From 1428 examinations, it was found that 28½ per cent. had tubercles in the lungs: nearly 2½ per cent. had tuberculous matter in the bronchial and cervical glands, and 8¾ per cent. enlarged and tuberculous mesenteric glands. Tubercle was more common among males than females.

"Of the males, nearly 36 per cent. and of the females, 21 per cent. had tubercles in the lungs; the proportion at different ages was as follows:—

| | Males, | per cent. | Females, | per cent. |
|---|---|---|---|---|
| Under 7 years, in | 147 | 23.83 | 147 | 14.28 |
| From 7 to 20 " | 24 | 29.1 | 32 | 25 |
| From 20 to 40 " | 132 | 58.4 | 112 | 39.3 |
| From 40 to 60 " | 180 | 47.8 | 156 | 25.6 |
| From 60 upwards | 203 | 22.1 | 295 | 15.9 |

"No satisfactory explanation has as yet been afforded why males should be more liable to pulmonary consumption than females; occupation alone is not sufficient to account for the great difference, as we find, from the above, the same disparity to exist even in childhood.

"*The weights of the internal organs in phthisis* were all above the average in both sexes. The disproportion was greatest in the lungs, which were nearly one-half heavier. The effect of age upon the organs seemed to be to diminish their weight.

"*The weight of the body of the male adult consumptive patient* was nearly one-third less than the average weight of the male adult in the workhouse; as the internal organs were all above the average weight, the great decrease in the weight of the body must have been in the muscular structure, tissues, and framework.

"*Height.*—The mean height of 107 male adult consumptive patients was five feet seven inches, and of 63 female consumptive patients, five feet two inches.

"The mean height of 160 female adult paupers in the workhouse, the majority from 35 to 50 years of age, was five feet and ¾ of an inch, and of 141 male adult paupers, a little more than five feet three inches.

"Hence, it appears that the consumptive females were 1¼ inch, and the consumptive males nearly four inches above the average height of other individuals belonging to the same class. It would be interesting to observe the relative height of healthy and consumptive individuals of both sexes, and of all ages, belonging to a different class, and occupying a different locality. Mr. Hutchinson, in a paper read before the Society of Arts on a pneumatic apparatus, has, from numerous observations on male adults of different classes, established a law—viz. 'for every inch of height from five feet to six, eight additional cubic inches of air at 60° are given out by a forced and full expiration.'

"Herbst found 'that adults of large stature, when breathing tranquilly, inspire and expire from 20 to 25 cubic inches; persons of smaller stature 16 or 18 cubic inches.'—(Vid. Müller's Phys. Part I, second edition, p. 313.)

"Does the rule laid down by Mr. Hutchinson, that persons, in proportion to their height, expire a greater quantity of air, afford any explanation of the foregoing results, that tall persons are more subject to consumption than short persons, under similar circumstances, and males more liable to the diseases that females? The greater depth of the chest and consequent length of lung, together with the specific lightness of the air, facilitating its accumulation and detention in the cells at the apex of the lung, in which part tubercles are most apt to collect, and become usually first developed.

"It deserves inquiry what may be the modifications of form—what the degree of elongation in the lungs of tall persons, and whether the air inspired under such circumstances may not render them more subject to the effects of any deleterious properties mingled with that air than shorter persons similarly situated.

"Of 60 healthy children measured—30 males and 30 females—from 3 to 7 years of age in the workhouse, the average height of the males exceeded that of the females by two inches, and the liability to pulmonary tubercles among the males at that age has already been shown, from 294 observations, to be more than 9 per cent. greater than among the females of the same age.

"The difference, as may be seen, is still greater after puberty, from 20 to 60 years, the period of life at which the males are obliged from their employments to use more bodily exertion than the females, and consequently to make greater demands on their pulmonary organs.

"As age advances, and consequent inability to exertion, the difference in degree to which the two sexes are affected is less even than it was in early life.

"The results respecting the relative frequency of phthisis in the two sexes, are directly opposite to the results obtained by M. Louis at La Charité; according to the observations of that distinguished physician, the frequency of phthisis among men in relation to women, was as 72 to 95.—(*Annales d'Hygiene,* t. vi., p. 51.) The fact is interesting, because it appears probable that the relative frequency of consumption in the sexes, of subjects nearly in the same class of life, differs essentially at St. Marylebone Infirmary, London, and La Charité at Paris.—*Dublin Medical Press.*

---

### A Medical Portrait.

Our Contemporary of the Dublin Medical Press, has drawn a portrait of the medical profession in England, which we, after forty years' experience, could not recognise, or, as Jonathan would say, *realize.* After alluding to the "comparative purity of the profession" in the Emerald Isle, "as contrasted with its condition in England," the reviewer proceeds:—"Here (Ireland) still, the body at large, of *physicians and surgeons,* is entitled to the rank of a profession—there (England) it is a trade, and, compared with other trades, not by any means the most respectable." The great body of physicians and surgeons in England will certainly be a little surprised—and not very much flattered by this information that they are only second chop tradesmen! "How can it be otherwise?" (says our polite contemporary) "into no other profession, business, or calling, in any degree entitled to be considered respectable, can a man obtain admission with less means and less general education. Whoever can scrape together about fifty pounds in the course of a few years, and can obtain his board and lodging for his work at a druggist's, while engaged in what is called his 'studies,' can, without difficulty, obtain a surgical diploma." Indeed! The Council of the College in Lincolns-in-fields will prick up their ears at this piece of news—and even the College of Apothecaries in New Bridge Street will stare at it. It is abundantly clear that our contemporary has travelled into a country of which he is perfectly ignorant; for we cannot believe that he had wilfully and knowingly stated as facts what every apothecary's apprentice here knows to be the merest and most ludicrous fictions.

### MEDICAL EDUCATION SCHOOL.

An establishment for the education of medical men's sons, whether designed for the medical profession or not, has been proposed by Mr. Martin, of Reigate, and warmly received by the Provincial Association. It is an admirable suggestion. Medical practitioners have often more difficulty in procuring good primary instruction for their children, than food and raiment for themselves and families. Such an establishment, therefore, would be an inestimable boon to a large portion of our hard-working brethren, especially in the country, where education is much more inaccessible than in the metropolis and large provincial cities. We think a general appeal to the whole profession, of all ranks and classes, could hardly fail of great success. Let energy, prudence, and liberality combine with strict impartiality in this great and good proposal.

### IRISH MEDICAL BENEVOLENT FUND SOCIETY.

No liberal profession suffers so much from temporary loss of health as the medical. The Bishop can order a substitute to do the duty of the sick clergyman, without injury to shepherd or flock. The attorney can direct his clerk, even when he is prostrate on the bed of sickness—but the physician, surgeon, and general practitioner lose their income the moment they are rendered incapable of personally attending to their patients! It is a wonder that some extensive association is not formed for raising a sum that may meet such melancholy contingencies. The Widows and Orphans Fund in London—the Provincial Association in the country—and various other local institutions, though productive of good, in proportion to their means, are quite inadequate to the relief of a hundredth part of the distress that is perpetually existing in the ranks of the profession.

The Institution at the head of this article is a meritorious one. The second Report lies before us, and informs us that—

"It is not intended either to be a Benefit Society, or an Assurance Club; but, strictly speaking, a Benevolent or Charitable Institution, founded and promoted for the express purpose of assisting our professional brethren, when struggling under the pressure of disease or other calamities. It is likewise proposed, under circumstances of peculiar urgency and distress, to administer relief to the widow or family of a professional man, who may have been deprived of the support and protection of a husband or parent. While, therefore, the sole design of this Society is to hold out the hand of charity and benevolence to a suffering and afflicted brother, or his family, it will not countenance improvidence or idleness, or evil habits of any kind."

The following are the chief rules of the Society.

"1. That a Charitable Fund be created by Donations and Subscriptions of Physicians and Surgeons to be called the Medical Benevolent Fund of Ireland.*

* "The Society will not acknowledge any one as a fit object of relief, who has not received a regular professional education, and is either a Doctor of Physic, or a Licentiate or Member of a College of Surgeons."

"2. That Contributions be received from all persons friendly to the objects of the Society, though not belonging to the profession.

"3. That the object of the Fund be the relief of Medical Men under severe and urgent distress, occasioned by sickness, accident, or any other calamity.

"4. That any Medical Man labouring under such afflictions be considered a fit object for the Charity.

"5. That the claims of Contributors shall, as far as possible, have the preference; but that contributions to the Fund give no claim of right to relief, the Fund being one of pure charity, and that each case be judged according to the urgency of the distress.

"6. That under circumstances of peculiar emergency, relief may be extended to the Widows and Orphans of Medical Men, it being understood that it is not the design of this Fund to relieve Medical Men from the necessity of providing for their families by ordinary life insurances, and such other means as prudence dictates.

"But if any provide not for his own, and specially for those of his own house, he is worse than an infidel."—1. Tim. v. 8.

"7. That the management of the Fund be conducted by Committees of the contributing members, annually appointed; the Central Committee to be at Dublin, and local committees, subordinate to the Central, in each of the principal cities and towns; the Central Committee having power to appoint local committees, wherever they may be required."

On the 22nd February a third meeting took place, Sir H. Marsh in the chair, and, from the report of the Secretary, the Association wears a thriving and prosperous appearance. The members of the profession in Ireland cannot too zealously support this young institution. We wish it every success.

---

### Northampton and Shire.

In an eloquent address to the Provincial Medical and Surgical Association, by that distinguished Physician, Dr. Robertson, of Northampton, there are many interesting remarks on the medical topography and diseases of that inland county. The shire itself is somewhat in the form of a wedge, the thick end of which (almost the highest table-land in the kingdom) is to the westward, while it slopes away eastward towards the Fens of Lincolnshire. From its great elevation it is very unfavourable to the pulmonary apparatus, and consumption is very prevalent and fatal. The habits and employments of the people, too, are unfavourable to health. In all the towns and most of the villages, the bulk of the artisans are busied in sedentary employments—the men in making boots and shoes—the women in binding them, and making lace. They are often huddled together in confined apartments, breathing a heated and contaminated air—or rushing out into the cool atmosphere in the evening, while jaded and exhausted. The effects of such habits, too often exasperated by beastly intemperance, may be easily conceived. Glandular obstructions, tubercular deposits, visceral congestion are the most prominent features of the diseases of the people here. The agricultural labourers escape but little more than the artisans, though their employments are so different. In them, the evil influence of cold and damp displays itself in the forms of rheumatism, dyspepsy, and disease of the heart, or acute rheumatism. Functional disorder of the stomach and organic disease of the liver are also frequent. All these Dr. R. refers to the keen air and damp. These when combined are extremely deleterious to the human frame. The subsoil of the greater part of Northamptonshire is a stiff clay that tenaciously retains the wet. The country in question is remarkably free from epidemics. Typhus, erysipelas, and the like, sometimes occur sporadically, but are never extensively prevalent. In 1828, ague was epidemic; but in that year it prevailed all through England. In that epidemic the inhabitants of higher grounds were more frequently affected than those of the lower. This has been observed in Tropical climates, and is evidently owing to the ascent of the miasmata, and their impinging against elevations of ground.

Dr. R. introduces an eloquent eulogy on the motives which draw the Provincial Association annually together, and the good effects which result from such meetings.

"The mere fact of our assembling in this manner, for purposes partly scientific and partly social, is infallibly productive of great benefits to the profession. It brings us together; it makes us personally acquainted; it enables us to know, to appreciate, and to esteem each other. It transmutes into harmony, right feeling, and mutual regard, those jealousies, estrangements, and heart-burnings, which have been too long the reproach, I might say the disgrace, of our noble profession. The Provincial Medical and Surgical Association is thus gradually bringing about a great and salutary reform; a reform, too, for which we are not indebted to the Legislature, nor obliged to court the smiles of any Minister of state. Gentlemen, I am second to no one in my anxiety for the real improvement of our profession; which, to say nothing of its rank as a science, is unquestionably to be classed as the noblest and best of the arts. But, although the honour, dignity, and prosperity of the profession, in all its branches, are dear to my heart, and although I do not undervalue discreet and well-considered reform, I have never been what is called a keen medical reformer. I have always felt that the greatest evils of the profession are such as no legislation can reach or remedy. It may be said of us, as has been said by some one, (I forget whom, for I quote from memory,) in a more general sense:—

"How many ills the race of men endure
That kings and laws can neither cause nor cure!"*

Dr. Robertson despairs (as well he may) of uprooting quackery from the land by any penal statutes; but this is a very different thing from throwing open the practice of medicine to every pretender, without enforcing a rigid examination upon all who undertake the cure of bodies, as well as of souls.

The Orator feelingly deplores the illiberality which the members of the profession too often exhibit towards each other: here is the grand field for reform. If we do not amend our conduct among ourselves, we need expect little from the Legislature. This address from the talented and excellent writer is replete with moral sentiments that do equal honour to his head and his heart.

---

### BIRMINGHAM ROYAL SCHOOL OF MEDICINE, &c.

By a letter from the Rev. V. Thomas to the Rev. Dr. Warneford, we perceive, with pleasure, that an educational and subsidiary establishment is about to be added to the Medical School, by which great improvements will be introduced

---

* We believe it ought to run thus:—

"Of all the varied ills that men endure,
How few are those that kings or laws can cure!"

Bad kings and bad laws (Napoleon and Cæsar for example) have *caused* innumerable evils; but they have *cured* very few of those to which human nature is liable!—*Ed.*

in the system of boarding, lodging, and private tuition, whilst the moral and religious education of the pupils will be carefully attended to. It is curious that this great desideratum should be first called into actual existence by a provincial town. The Schools of London, Edinburgh, Dublin, are all destitute of a collegiate establishment, where the health, morals, and religion of medical students might be watched over. The scattered, isolated, and uncontrolled manner in which young medical pupils live, is too often destructive to mind, body, and professional prospects in after-life.

There will be one great difficulty, however, in the formation of such a collegiate establishment, whether at Birmingham or elsewhere—RELIGIOUS CREEDS! "This, as well as all other regulations appertaining to religion, should be based upon the doctrine and discipline of the Church of England, one of whose Ministers ought to be the resident Warden or Principal of the College." Here is the difficulty! The Catholic, the Dissenter, the Israelite who choose to study medicine, cannot avail themselves of the discipline and protection of the collegiate establishment, and must shift for themselves! This is, and is likely long to be, the great stumbling-block in the formation of colleges attached to Medical Schools. It is one over which the London "UNIVERSITY COLLEGE" itself could not get, with all its talents and ingenuity!

---

### MALARIA—TEETOTALISM.

Mr. Morris, who practises at Spalding, in Lincolnshire, states that, from observation among the fenny and malarious parts of that country, the TEETOTALERS are more liable to suffer from malarious affections, as fever and intermittents, than those who take a moderate portion of stimulants. This is consonant with universal experience in all unhealthy climates, whether inter or extra-tropical. If, however, moderation in vinous or malt liquors be exceeded, the opposite effects are produced. The excess predisposes to malarious impressions even more than the total abstinence from stimuli. Mr. Morris illustrates his proposition by allusion to Irish labourers (Teetotalers) who come over in the Autumn, and who are apt to get ill till they break Father Mathew's pledge, and drink some English stout.

---

### UNITY OF SENTIMENT.

Our respected cotemporary of the GAZETTE, thinks he has at length discovered unity of sentiment in the medical profession from the Pentland Frith to the Cliffs of Shanklin—from the Lizard to the Goodwin Sands—REPROBATION of the "New Medical Reform Bill." But our contemporary is rather premature. The Dublin "MEDICAL PRESS" has taken up the cudgels in favour of Sir James Graham's "free trade in physick," and Dr. J. Davies, of Hertford, has stept into the field, as a valiant Lieutenant to the Hero of the Green Isle. With the latter advocate of things as they are to be, we shall not meddle; and with the columns of Dr. Davies in the Provincial Press, we shall not occupy much space. The gist of the Doctor's arguments lies chiefly in this fact—that legal restrictions, pains and penalties have not, hitherto, been found equal to the eradication of quackery—and, ergo, such pains and penalties should be erased from the Statute-book, as disgraceful to modern freedom in trade. All that we shall say in answer to this is—that as the pains and penalties—including a swing on "Tyburn tree"—which have been enacted against MURDER, have not, hitherto, erased that foul crime from the long lists of our criminal calendars—ergo, all punishments for such offence should be sponged away from our legal tablets!

Did it never occur to our sapient doctor, that although crimes cannot be completely eradicated by human or even Divine laws, they may be checked and lessened by them; and that, at all events, they ought to be held up to general reprobation by penalties however inadequate to their entire prevention.

But it is not open undisguised quackery alone that should come under the operation of legal enactments. Sir James Graham's "voluntary system" of examination and registration will let in a flood of half-educated practitioners who will do more harm to the practice of physick and surgery than all the quacks of the land. This is THE evil of the contemplated measure!

### DR. DALTON.

It is an old and trite remark, that the lives of literary, scientific, and philosophic men seldom present such important epochs and events in their mortal career as to greatly interest the general mass of mankind. The Nelsons, the Napoleons, the Wellingtons, whose genius or talents decided the fate of battles, or even of nations, are the grand subjects for popular biography, and their eventful lives are sure to fix the attention of all readers.

The late Dr. Dalton, of Manchester, the father of our chemical philosophers, of modern times, occupied no small space in the scientific world, and has recently paid the debt of Nature at the mature age of 78 years. He was born at Cockermouth in September 1766, and little is known of his early history, except that he was fond of mathematics. Between 1784 and 94, his name appears as a frequent contributor to the Gentleman's and the Lady's Diary. In 1788 he commenced his meteorological observations, which were continued to the day of his death. In 1799 Dalton began to teach mathematics and natural philosophy. A small circuit round his laboratory, in George Street, appears to have been the limits of his peregrinations for many many years. The Memoirs of the Literary and Philosophical Society of Manchester have presented almost unbroken records of Dr. Dalton's labours till nearly the close of author and books. The greatest of his discoveries was the atomic theory—1803. His whole life seems to have been spent in teaching chemistry; but the longest life will come to an end. In April 1837, Dr. Dalton experienced an attack of paralysis, which deprived him of the use of one side, and also, for a time, of speech. After this, he had repeated attacks, together with other illnesses, which greatly impaired his physical strength and intellectual energy. On Saturday, the 26th July, he appeared in his usual health, but in the night breathed his last, without a struggle or a groan! The Manchester Guardian says:—"Science has lost one of its most devoted sons—England, one of its greatest scavans—and humanity, one of its brightest living examples of the wisdom of the philosopher, united with the purity and simplicity of the child."

### FREE TRADE.

If Ministers have been unable or unwilling to throw open a free trade in Corn, they seem determined to do it in Physic! The MILLENNIUM MEDICUM is fast approaching. In the year of grace, 1845, *unlawful* medical practice will cease throughout Great Britain. In that year all *preventive* checks on the noble art and science of medicine and surgery will vanish into air—thin air! We do not allude exclusively to *Charlatans*. They have always been respected by the community at large in this country, and are now to be under the especial patronage of the Legislature. But we look to the half, or the uneducated medical practitioner, who will be able to open shop in New Bridge Street, Black-friars, under the very nose of the worshipfuls, and prescribe and dispense medicine without let or hindrance! But Sir James Graham tells us there is to be a *penalty* on unlicensed practitioners. It is certainly an Irish one. Such practitioners are not to be *rewarded* for their ignorance or want of diploma! They are not to be promoted to places of rank or emolument. They will not be eligible (they are not so now) to appointments in Hospitals, the Army, the Navy, &c. Why no. They would take good care to decline such appointments were they offered to them. They covet no such theatres for the display of their abilities. They will be perfectly content with the liberty of starting in every street of the metropolis, or of provincial towns, to practise medicine, surgery, midwifery, and pharmacy, quietly, *legally*, and lucratively, without any disturbance from the beadles of a College, or the solicitors of a trading company. The Home Secretary tells us that the great principle of his Bill is to encourage rather than repress—to offer rewards for knowledge rather than impose penalties on ignorance. Very fine words; but the effect will be just the reverse. The working of the Bill will tend to foster ignorance and effrontery—to depress skill, learning, and science. It is hesitatingly proposed to deprive the unregistered practitioner of the power of recovering charges in a court of law. What cares the quack or the ignoramus for this? Do we ever see the Charlatan go to law for the recovery of his medical debts? Never. Nor is this boon of any use to the regular practitioner. He ought never to go to law with his patient. He will lose ten times more than he will gain by such a procedure. And, after all, can any physician in Great Britain recover a fee? Not one; and physicians thrive as well as other classes of their brethren.

The grand feature, or principle, if you will, of this Reform Bill is, to give every man the free and legal power to practise medicine, in its most comprehensive sense, whether qualified or not—and, at the same time, offer to the public full scope and liberty to employ the unlicensed practitioner, if it pleases, in preference to the qualified and registered. It is very true that the public cannot be prohibited from consulting quacks and risking their lives; but this is no reason why quackery should be encouraged by Government, and all restraint on its operations taken off. We cannot prevent a man from purchasing poison and destroying his own life by it; but we can punish a man for vending that poison incautiously, and thus facilitating the act of suicide. This is the plague-spot on the new Bill; for the centralization of power in the Council of Health, and the equalization of education, fees, and rights of the different colleges, are decided improvements on the old and heterogeneous system of legislation.

"Adversity makes us acquainted with strange bed-fellows." It is not a little curious to observe that the Worshipful Company of Apothecaries, which has been nick-named, scorned, and scandalized for twenty years past by the ultra-reformers, has, all at once, found favour in the sight of their enemies, who have suddenly discovered virtues and merits in the "hags of Rhubarb Hall," which never radiated on their optics till the new Reform Bill made its appearance!! The said Reformers see at last that the "old hags" have done great good in their day, and that they are now most shamefully treated by the Government. They, therefore, hold out the hand of good-fellowship to the "Weird Sisters" of Blackfriars, and offer to stand by them till the death, in resistance to that odious and cruel tyrant—the Græme Macbeth!

So then, the Medical Agitators in this Country have had little more success than the Repeal Agitators in the Green Isle. The latter had got a comfortable "Locus Penitentiæ" on the banks of the Liffey, while the former are granted a Tempus Penitentiæ of some ten or twelve months, during which they may chew the cud of disappointment at leisure! After a twenty years' struggle for a representative, or rather a fœderal government—a great tripartite faculty—a triple-headed Cerberus to watch the interests of the three branches of the profession—we are to have a great hydra-headed monster, appointed, not elected—and armed with powers over which we shall have no control. We were dissatisfied with King Log—and now we shall have King Stork! When we cast a retrospective glance at the juvenile days of Medical Reform, and remember the thrilling harangues of a Lawrence and a Wakley in Freemasons' Hall, we cannot contemplate the present scene—the sad result of all their exertions, without exclaiming in the words of the immortal Bard—

——————— though far away
Each flower that hail'd the dawning of the day,
Yet o'er her wither'd Hopes that once were dear,
The time-taught spirit, pensive not severe,
With many a tear her aged eyes shall fill,
And mourn their falsehood, though she love them still!

It is clear that the Home Secretary has based his Medical Reform Bill on the new system in Hanwell, where all pains and penalties are abolished, and every thing is to be done by coaxing and kindness. He, no doubt, views quackery itself as a species of refined philanthropy, where the good of the public—the salus populi—is alone considered, and no regard paid to private pelf. Who would be so ungenerous as to suppose that Morrison recommended his pills for any other purpose than to save the lives of thousands, and that without the slightest view towards the accumulation of wealth? Under the new system the Morrisons and the Longs will be no longer harassed by law-suits or trials at the Old Bailey. The prospect of a professorship in one of the colleges or hospitals will be quite sufficient to discourage or suppress the most inveterate quackery!

That one supreme or ruling Body should exist in the metropolis for regulating the affairs of the profession, and equalizing the various licensing colleges and corporations throughout the empire, no reasonable man can deny; but the *composition* of that Council requires great care. The *ex officio* members are, doubtless, good men, but we fear that interest more than merit will operate in the appointments by the Crown.

And yet a few strokes of the pen—one or two clauses added—and two or three struck out, might render this stigmatized Bill as good as we can, under all circumstances, hope to obtain. In the words of Goldsmith, it might be said of this enactment, that—

"It has but one fault—but that is a thumper."

A clause rendering the registry (after proper examination) imperative, not optional—and imposing a penalty on all those who practise without that registration, would make the Bill very useful. With such clause the mock incentives of hospitals, public appointments, &c. &c. may be struck out, or left to find their own level, among clashing interests and the struggles of competition.

---

### Influence of Locality on Disease.

Dr. Rumsey, of Henley-on-Thames, has called the attention of the profession [*Prov. Med. Journal*] to the peculiar salubrity of Beaconsfield, near High Wycomb, Bucks, which, if his representation be correct, would appear likely to prove the Montpellier of England. Dr. R. is of opinion that a "noxious and unsuspected element lurks in our soil," whose solution in the waters poisons the blood, and affects various organs of the body. He thinks that this poison prevails much more in some places than in others. Our author has long believed that BEACONSFIELD is distinguished for the salubrity of its air and water far beyond other neighbouring towns and villages. Yet the elevation is not great—the soil is none of the driest—and the temperature of the air is far from warm. Nevertheless, this hill is exempt from several diseases that prevail in other and apparently more favoured localities.

"In the numerous surrounding towns, which are for the most part situated in vallies, some wide and open, others comparatively narrow and contracted, bordered by hills of chalk, with superincumbent gravel, clay, or sand, yet of drier soil and houses than Beaconsfield, I have met with much more articular and glandular disease. More persons by far are the victims of pulmonary consumption; the stiff and useless joint, and cripple, are seen with much more frequency; the goitre is met with at every town; calculus of the bladder has been seen in most, if not in all of the other towns and villages of the neighbourhood, and that of the kidney is very common; whereas, the former of these, calculus of the bladder, I have every reason to believe, from the authentic source of my father's long and extensive experience in this vicinity, and my own opportunities of observation, has not been seen at Beaconsfield in a single instance for more than half a century, and possibly never to have arisen there, and the latter, gravel, is only exhibited by a rare exception in the corpulent, the bed-ridden, and the sufferer with disorganized kidney.

"The goitre itself may be said to be unknown in this town, though not three miles from one of its favourite localities, and absolutely surrounded by districts fruitful in its production.

"Other painful affections, incident to the young female, yield to the same law of comparative exemption in this favoured elevation, and my conjecture is, that these advantages do not complete the whole catalogue which belong to this town's distinguished geological position."

"I have witnessed the spontaneous cure of diseases on a change of abode to this hill, which usually, under other circumstances, make a very different progress. Dysmenorrhœa has been removed; bronchocele has disappeared; attacks of gravel have permanently and spontaneously ceased; and, in one instance, the gradual, yet speedy, total and permanent cessation to cough up chalk stones, followed a residence at Beaconsfield, the native and previous abode of the patient having been one of the surrounding localities, more fertile in disease."

Medical topography is an interesting and valuable study. It is a pity that such investigations are not more frequently pursued than they are. Dr. Rumsey has related some curious cases illustrating the prophylactic and sanative influence of Beaconsfield, which we recommend to the attention of our medical brethren in that vicinity.

---

### KINGDOM OF SHOA.

It appears that England has an embassy at the capital of this small kingdom in Abyssinia, situated near the Straits of Babel-Mandel, and whose medical topography has been drawn up and published by the Surgeon to the Embassy, Rupert Kirk, Esq. in the Bombay Medical and Physical Transactions. The territory lies between eight and ten degrees of North latitude, and 38 to 40 East longitude. It consists of two table-lands—the lower, 2500 feet above the level of the sea—and the upper rising from eight to ten thousand feet. The climate of this region is highly-favoured. ANKOBER is the capital—the residence of the Embassy—and containing some ten or twelve thousand inhabitants. The upper plain possesses a pure and buoyant air, and though close to the Equator, the tropical temperature is greatly reduced by the elevation of the soil. The thermometer ranges from 70 to 75 in the day, while the nights are chilly, and thin pellicles of ice are occasionally seen in the colder months. The mean annual temperature of Ankober is 55½—three degrees lower than that of the Neilgherries on the coast of Malabar.

"Perhaps there is no kingdom which possesses a greater variety of climate within its limits than Shoa. Leaving the parched plains of the Adiel on which is felt the fierce heat of tropical Africa, a journey of eight miles brings the traveller to Farri, 3000 feet above the sea, and possessing the genial warmth of an Indian province; proceeding onwards twelve miles, he reaches Alioamba, which with an elevation of 5000 feet, enjoys a mild, Italian sky; from whence ascending the Ankober mountain, a ride of but five miles, he gains a height of 8,000 feet, and a climate only to be compared to an English spring, whose coldness may be judged from the fact, that fires are needed in the house morning and evening throughout the year."

This being the case, we should not be surprised to find Shoa become a SANATORIUM for our tropical invalids, dividing the spoil with Neilgherry and the Himalayas. The population of Shoa is calculated at about two and a half millions, of whom one million may be considered as Abyssinian Christians—the others are chiefly Mahomedan. The Christians are tall, stout, and well-formed, and varying in height from 5 feet 7, to 5 feet 9 inches—some of them are upwards of six feet. Their colour varies from brownish olive to the clear black of the Negro. The most common colour, however, is a coppery brown, with a tinge of black.

The food of the Abyssinians is chiefly farinaceous, and the meals, as in India, taken about sun-rise and sun-set. Their drink is a kind of mead, prepared from honey and corn, and also tulla or beer brewed from barley. It appears that these people are subject to almost as many diseases as our polished and civilized nations, as the list of maladies, for which Mr. Kirk was consulted, will shew. Three hundred and seventeen syphilitic cases, among others, were treated by our author! The tænia solium is also very prevalent. Very few of the natives are free from it. They attribute it to the eating of brindo, or raw flesh. Fortunately the flowers of the Kosse, a tree common in Abyssinia, are an effectual remedy for the worm. It is taken regularly every two months by the inhabitants. Its operation often produces prolapsus ani—being a drastic purgative. Scrofula is very common among the females, as is also bronchocele;—fever, though formidable, is chiefly prevalent among the low and fertile valleys, where water is abundant. Epilepsy, and other nervous affections, are sufficiently common. There are no professed doctors—medicine being strictly domestic. All ranks attempt to secure themselves against diseases by amulets or charms—and these are probably as successful as the articles in the family medicine-chest among ourselves! "Should a person be attacked with fever, the head of a red cock is sometimes cut off, with certain rites and formalities, and its blood is sprinkled on a road, when they believe that the disease will be transferred to the first person who crosses the infected spot." This is a very generous and disinterested practice! Surgery is on pretty much the same level as physic, which is not saying much for that science.

---

### American Notes.

Many persons in this country are calling on the Legislature to put down quackery by law. Whether law could succeed in doing so, we will not stop to ask. The lawgivers of Alabama once thought it would, so they went to work in earnest. Their success would not seem to be encouraging, for we read in a Journal of the West:—

"Alabama has long had a law denying to those who practise medicine without a diploma or a license, the benefit of her courts in the collection of their debts. We do not suppose it has done any more good in this than in other states; but still it served as a theoretical expression in favor of science. Lately, however, it has been so modified as not to interfere with 'any persons' who practise on the 'botanical system of *doctor* S. Thompson;' provided, nevertheless, that they 'do not bleed, apply a blister of Spanish flies, administer calomel or any of the mercurial preparations, antimony, arsenic, tartar-emetic, opium, or laudanum!' It would be difficult to say whether *doctor* Thompson's patent, or this law, is the more precious specimen of empyricism. It is marvellous, that a people so enlightened as those of Alabama, should allow their statute book to be made ridiculous by such nonsense."

It is pretty clear that the enlightened folks of Alabama got sick of being doctored only by the faculty. They tried it, but it did not suit them, and so Doctor Thompson turning up with his botanical system, they made an exception in favour of *him*. Bye and bye Dr. Thompson and the botanical system, too, will be found or fancied a delusion, and some one else with a wet system, or a dry system, or some system of some sort, will be taken up in his turn. And this is the history of the human mind, whether in the old world or in the new, in the aristocratic halls of London or the forests of the bowie country. When legislators can be found who are not to be caught by quackery or quacks, then we may think of law, but not before.

### MESMERISM IN THE FAR WEST.

We all know how very scientific men have taken up Mesmerism here. This grand discovery has been accepted by the titled and the scientific, and it cannot be said that a deaf ear has been turned to its marvellous tales by the choice spirits of the land. The Marquis of Anglesea has smiled upon it, Doctor Elliotson has lectured on it, and half the aristocracy have half believed it. We are not then altogether a nation of infidels, and faith has a resting-place amongst us. But in Alabama, Mesmerism has grown downright vulgar. It is positively low.

"Alabama is not more thoroughly overrun with the disciples of *doctor* Thompson, than those of Dr. Mesmer. Anxious to have the phenomena of Mesmerism subjected to a rigid examination, the true separated from the false, and the public mind kept in a healthy condition in reference to both, we cannot but regret to see one travelling mountebank after another, traversing the south-west, for the purpose of extracting money from those who are credulous enough to believe, that *he* can do any thing in which men of sense will confide. It is truly unfortunate for Mesmerism that it should have fallen into such hands. Things are coming to that kind of pass, that we shall soon not be able to distinguish the pass of an impostor from the pass of a scientific Mesmerizer, and, in discouragement, pass the whole by; not even making it a pastime of a passable kind, as it was in days past; and when this happens, there is great danger that it will be passed over, and fall into a somnambulic state, from which the passes of the most scientific hand may not awaken it, till the remembrance of what is now doing to degrade it has past away."

We do not know if all these not very passable puns are meant for Mesmerism or the Mesmerizers. But however that may be, one does not like to see a science so exalted get into such low hands. We dare say the fellows Mesmerize for half a crown. Just conceive somnambulism, reading with one's back, the gift of prophecy and so forth, disposed of for the small charge of two shillings and sixpence. It is quite distressing. Oh! Dr. Elliotson what *are* you about. We hear of missions to China. We would suggest to the Doctor to get up one for the poor deluded people of Alabama.

### CAPITAL NEWS FOR YOUNG DOCTORS.

We really cannot wait to talk about the matter, but communicate the following "important news" to our young friends. *Won't* there be a rush to Alabama.

"In the north and west every village has its old doctor—generally quite a respectable personage, with several peculiarities, and the self-complacency which comes from a life of usefulness, winding up amidst the regard of the neighbourhood, and the respect of the younger physicians, who surround him. A sage of this kind is almost unknown in the south-west, except by title, which often indicates about as much clinical labor, as that of colonel testifies to military achievement. The reason of this difference between the south-west and the more northern portions of the Union, is three fold:—1st. A great many physicians die young; 2d. A number go to cotton-planting; 3d. Not a few marry rich widows. Thus it is that causes the most opposite conspire to deprive this quarter of the benefits of ripe medical experience. How long the first will continue with its past energy, we cannot predict; but the second will soon cease unless the price of cotton should rise above five cents a pound; the last, however, is of a permanent character, for six or eight times as many husbands as wives die in this region. We shall expect the thanks of at least two classes of persons for this information."

We should think so. What a good fellow this Editor is. And a close reasoner too. What, for instance, can be more certain than that if doctors die young they won't live to be old. They should work double tides in Alabama. For, first of all, they have the chance of dying youug as Doctors, and, secondly, if they are not thick enough to do that, they have another chance of dying young as cotton-planters. Nay, when they marry they are pretty certain to die before their wives at last. They thus realize the somewhat groggy idea of Alexander, who, in his cups, "thrice slew the slain." The great requisite for emigrants to Alabama is to have the lives of a cat.

### Death in the Fire-Place.

Most people recollect the alarming work of the respected Mr. Accum—"Death in the Pot." Such as read it must have wondered how they ever contrived to live so long, indeed to live at all, every article of food, being proved to be deleterious.

Now, however, the matter is shewn to be worse than even Mr. Accum made it out. Not only are we eating and drinking slow poison, but the air we breathe in our bed-rooms and sitting-rooms is next to certain death. An ingenious writer in the Medical Gazette puts this in such a striking point of view, that the firmest nerves must be shaken by it.

"It should be borne in mind," says he, "that the openings of our fire-places being seldom more than three or four feet from the floor, the upper stratum of air is neither removed or purified by this under current, and must, from being breathed over and over again, be productive of most prejudicial effects, and that this contamination of the atmosphere is considerably augmented at night by the combustion of lights, the quantity of air breathed by an ordinary-sized person being calculated to be about 2000 cubic feet per hour, and that two mould-candles consume as much of the oxygen of this air as a human being, the nitrogen and carbonic acid gas which remain being peculiarly inimical to animal life, and that when carried up by the currents occasioned by combustion and respiration, they must be repeatedly inspired in this upper stratum before they make their escape into the chimney—the only ventilating flue with which our houses are provided; that the heat thus generated is in proportion to the quantity of oxygen abstracted from the atmosphere, which enters into combination with the carburetted hydrogen of the flame of candles, coal, gas, oil, or other inflammable matter from which light is produced; that every cubic foot of carburetted hydrogen consumed unites, on an average, with two cubic feet of oxygen (that portion of the atmosphere required to support animal life); and that the product of this combustion is about two inches and a half of water and one of carbonic acid gas, which, when inhaled in its pure state, proves instantly fatal; and the greater the proportion we inhale in addition to the animal vapours evolved from the lungs and skin, the more pernicious the effect.

"Supposing, for example, that the perfect lighting of an ordinary sized apartment requires fifteen cubic feet of carburetted hydrogen per hour, this would form about a pint and a half of water and fifteen cubic feet of carbonic acid gas, which are the products of the combustion, whether the carburetted hydrogen is obtained from wax, tallow, oil, or coal. If, therefore, the lighting continues in an unventilated apartment for seven hours, one gallon of water is produced, the greater part of which must be deposited on the walls, windows, furniture, polished metal, or other cold surfaces with which it comes in contact; and to some articles of this nature it is known to prove highly prejudicial, in addition to the injury to health occasioned by an increased quantity of moisture mixed with the air we breathe. As one of the principal functions performed by this air for the preservation of health is to carry off with it a considerable quantity of vapour, in order to prevent its undue accumulation on the lungs, it is therefore evident that after it has been already so loaded it cannot properly perform these functions, and that consumption and other complaints are thus frequently induced."

We can only say, as we have said before, that the wonder is how we get on at all. With nothing genuine to eat—only deleterious mixtures to drink—and nitrogen, carbonic acid, and carburetted hydrogen to breathe, it is next to a miracle that any of us see forty. One reflection, however, springs out of this, which is a comfortable one—if we contrive to hold on to sixty, seventy, and eighty years, in the midst of such deadly agencies, what may we not hope to do bye and bye—when, thanks to chemistry, death is no longer in the pot nor in the fireplace—when bread and beer cease to be adulterated—when candles no longer furnish arsenic—when our rooms are not filled with nitrogen, carbonic acid and so forth. The age of Old Parr will then be the rule, not the exception, and the stunted race which now haunts our streets like melancholy ghosts, will be changed, under the influence of pure air and aliment, into something more like to men. The sooner the better, say we.

---

### Scalp-Seaton.

This remedial agent, in cerebral affections, is now "going the round of the press," to use a common expression. Dr. J. Johnson has, for more than thirty years, been in the habit of employing this species of drain in epileptic and other cerebral maladies, and has repeatedly noticed it in the pages of the Medico-Chirurgical Review. Dr. Johnson's method is more simple, and less painful than that which has lately been proposed. It consists merely in drawing a line of the kali purum along the course of the sagittal suture,—poulticing till the slough clears away—and then inserting a few threads of silk or cotton daily, imbued with the ceratum lyttæ. A purulent drain is thus established, with very little trouble, and with great benefit in obstinate cerebral affections. Mr. J. Johnson, of Rickmansworth, wore a seton of this kind for some years, under Dr. Johnson's direction.—30*th September*, 1844.

ABDOMINAL HYDATIDS. (Ed. Monthly Journal, August.)

"Dr. Gairdner read a remarkable case of abdominal disease, in which he had twice withdrawn by paracentesis from the peritoneal cavity, a quantity of a glutinous substance, resembling in colour and consistence calf's foot jelly, and coagulating, like the albumen of eggs, by heat. Some of this matter Dr. G. had exhibited to the Society in January, but in a comminuted state, in consequence of having passed through the canula, and the valves of the syringe adapted to it for suction. After the death of the patient in February last, it was ascertained that the whole abdomen was filled with similar matter, amounting in all to about twenty-four imperial pints, and that it consisted of rounded masses having very much the same outward appearance as the ordinary acephalocysts of Laennec, but differing from them in many important particulars. These remarkable parasites had invaded the textures of the liver and omentum, as well as of some of the other viscera. The right lobe of the liver was entirely destroyed by them; the left partially; the omentum converted into a dense and almost cartilaginous tumor, of large size and great thickness, in which many of the parasites were contained, and from which many of them hung pendulous. Dr. G., after detailing the facts of the case, gave a condensed account of the results of his inquiries into the literature of the subject. He found that extensive destruction of the liver by hydatids was by no means uncommon, and that there were one or two recorded cases, and more especially a very remarkable one by Ruysch, in which the destruction of this organ appeared to have been equally extensive as in his own case. He had not been successful in finding any case in which the omentum had undergone a change of the same sort precisely with that which he had described. Finally, he had examined attentively all the systematic treatises on entozoa in general, or on hydatids in particular, which were accessible to him, besides a great number of pathological works and individual cases in which they were incidentally described, and he had not yet found a single description of a parasite possessing the same physiological and structural peculiarities. He was therefore of opinion, that it was a rare one; and that, although it had in all probability been occasionally seen, its peculiarities had not yet been observed or described."

*Examination of the above Entozoon.*—"Mr. Goodsir read to the Society an account of the structural and physiological peculiarities of the entozoon found in Dr. Gairdner's case, by Mr. Henry D. S. Goodsir. Mr. Goodsir, after quoting from his brother's paper, read to the Royal Society a description of the animal in question, explaining more particularly the structure of the parasite,—stated as its most distinctive peculiarity the fact, that instead of being like the common acephalocyst, a simple animal, it might be regarded as being of a composite nature. It appeared to consist of several cells, having in their interior a glutinous matter contained in cellular tissue, connected together by a common membrane, extending from the free surface of the peritoneum over the surface of the various cysts, and forming their pedicles. On the surface of this membrane, except on the part covering the globular cysts, there were seen numerous circular discs, around the margin of which were a series of stomata opening into tubes extending into the substance of the membrane. These Mr. Henry Goodsir regarded as being the nutritive organs of the animal. With regard to its reproduction, Mr. Goodsir remarked, that this took place in two ways, one which respected the extension of the individual existing group, the other as regarded the propagation of the animal to uninfected tissues. This, like the common hydatid, did not enlarge from cellular development, but by simple expansion of the original germinal vesicle. As regards its propagation to uninfected tissues, Mr. Goodsir stated, that he had been unable to trace the earlier process by which an ovum appeared in the healthy tissue, but he found that, when this had occurred, the ovum enlarging makes its way like an abscess to the free surface of the organ, and bursts, leaving an orifice leading into a cavity of the tissue. The formation of these cavities gave to the peritoneum the honey-comb appearance, observable in Dr. Gairdner's preparations. After the bursting of the sac, the animal is not discharged from the cavity, but remains attached by its common membrane to the base of the cavity, and then projects outwards, elongating its outer membrane so as to form a pedicle."

---

### Uterine Diseases.

From a practical paper read before the Edinburgh Medico-Chirurgical Society lately, by Professor Simpson, on mechanical dilatation of the os and cervix uteri, we extract the following passage :—" Lastly, Dr. Simpson offered some observations on the introduction of the sponge tent into the os of the pregnant uterus, in certain conditions in connection with abortion, and as a means of inducing premature labour. When abortion was inevitable, and the hæmorrhage great, a small expanding sponge tent passed into the os uteri was more effectual than a large vaginal plug. It at the same time opened up the os uteri, so as to allow of the more easy escape of the contents, whilst uterine contractions were, in most instances, ultimately induced by its presence. For the same reasons it was often a valuable means of both opening up the os uteri, and exciting the necessary degree of uterine action in those occasionally perplexing cases where, in abortion, the embryo escapes, but the secundines are long retained. Dr. S. had employed the same simple means in inducing premature labour, and spoke of the advantages of it in comparison with the various other measures that had been proposed for that object. He found that the tent, when made and introduced in the mode already stated, required no vaginal plug, or other means to hold it *in situ*. By its use the first stage of labour, or the dilatation of the os uteri, could, in a great degree, be advanced, before the labour itself actually began."—*Dr. Cormack's Journal, Aug.* 1844.

---

### Malaria.

A Reviewer of Dr. M'William's "Medical History of the Niger Expedition," in the Athenæum, having doubted the existence of Malaria, attributing what are called malarious diseases to other causes, as the "ordinary accidents of climate, heat, and humidity," Dr. M'W. combats the Reviewer's scepticism by a paper in the same journal, for 21st September, 1844.

We suspect that the reviewer had never practised in a tropical or in any malarious climate, else he would not have considered miasmata, malaria, marsh effluvia, or whatever name we may give the poison, as a creature of the imagination. The following quotation from Dr. M'William's "reclamation," must be satisfactory to most of our readers, though ten thousand other instances and facts equally stringent might be adduced in proof of a morbific emanation from certain soils, exclusive of heat and moisture.

"Heat and moisture are conditions of the atmosphere which readily admit of minute quantitative determination, by methods in common use; and if fever were caused by them alone, in Europeans within the tropics, it should prevail wherever their amount is the same. Now, by reference to the meteorological tables in my work, the temperature and dew point outside the Niger, where no fever occurred, and while in the rivers, were as follows :—

| | Temp. 3 P.M. | Dew point, 3 P.M. |
|---|---|---|
| Passage from Sierra Leone to Accra .. .. .. | 81·13 | 74·93 |
| Outside Niger from 9th to 12th August .. .. | 79·00 | 73·70 |
| In the Nun and ascending to Aboh . .. .. | 80·60 | 74.00 |
| At Aboh, Idduh, and the confluence of the Niger and Tehadda to Sept. 21 .. .. .. .. .. .. | 84·00 | 73·50 |
| Confluence of Niger and Tehadda to Egga.. .. | 86·60 | 72·00 |

"Thus, though the expedition was exposed from the 1st of July to the beginning of August, to air containing more moisture, and but little inferior in temperature at the hottest part of the day, to any experienced within the river, not a case of fever made its appearance until the 4th of September, three weeks after it had entered the river, and had been exposed to the emanations from the ordinarily recognised sources of malaria. Similar results have been observed elsewhere; in Barbadoes, for instance, no fever occurred among the troops in the garrison during August, September, or October, 1841, and although in November a very violent description of yellow fever broke out, the temperature of the air was lower than in August, and the dew point lower than in September; their means were as follows:—

| | Temp 3 P.M. | Dew point, 3 P.M. |
|---|---|---|
| August.. .. .. .. .. | 83·77 | 70·61 |
| September .. .. .. .. | 82·13 | 73·78 |
| October .. .. .. .. | 82·31 | 72·67 |
| November .. .. .. .. | 82·83 | 71·67 |

"Hence the connexion between 'heat and humidity' of the atmosphere and severe remittent, or yellow, fever is by no means so clear as the reviewer would have us suppose. It is, in fact, one of those hasty conclusions which will not stand the test of comparison with observed facts, and could only have been made with a limited view of the history of disease in warm climates.

"At Barbadoes the fever was almost completely confined to one of the regiments composing the garrison, while the other, the men of which were equally exposed to 'heat and humidity,' and performed the same duties with their neighbours, was almost wholly exempt. The cause of the disease in this instance, was very obviously the effluvia arising from a pool of water, immediately to windward of the building occupied by the regiment that suffered.

"But to return to the west coast of Africa. In 1836, H.M.S. *Scout*, under the command of Capt. Robert Craigie, proceeded to the west coast; and by a careful observance of the stringent 'General Orders' of the senior officer on the station, 'that no ship was ever to remain in port more than forty-eight hours at any one time,' and that officers were so far as was practicable to avoid entering any of the rivers on the coast,' only two cases of fever occurred in her during the first year, and these were traced to two days' stay at Sierra Leone. In the month of April, 1837, Capt. Craigie was obliged to ascend the Bonny river, in the *Scout*, as far as King Peppel's town, for the protection of the British mercantile interests there. On this occasion, he also took the *Dolphin*, a brigantine, with him, and left the *Lynx*, another brigantine, anchored at the river's mouth. The *Scout* and *Dolphin* were detained nearly a week at Bonny town, and on leaving the river fever broke out in both vessels, and their united loss by death amounted to five officers and seventeen men and boys, while on board the *Lynx* not one was even attacked. Bonny town is only about six miles from where the *Lynx* was lying, consequently there could have been very little, if any difference as to the 'heat and humidity' of the atmosphere in the positions of the vessels that suffered and that which escaped.

"Capt. Brunswick Popham commanded the *Pelican*, with a complement of 110 white men, for four years and a half, on the east and west coasts of Africa.

During this time, his loss by death amounted to three Europeans. He made it a rule to avoid rivers, his boats having on one occasion only been in the Bonny, and that for a very short time. Capt. Popham was on the station during 1835-6-7-8, and part of 39, during which the mortality on the coast is but too well known. In short, it seems to me perfectly clear, from the evidence of many old African cruisers, non-professional as well as professional, and from my own experience, that, as a general rule, a ship will continue healthy on the west coast of Africa, if she is clean internally, and keeps at sea, and that disease will appear if she remains much nearer the shore, or has intercourse with the rivers. If we admit the immunity in the one case, and the occurrence of disease and death in the other, surely the destructive agency must have been owing to something connected with the land, which is acted upon by the same meteoric agencies as the sea, with this difference, that the land and sea breezes become more feeble as we advance into the interior. The sun is mainly effective *from below* in heating the atmosphere on land and water, both of which absorb its rays and communicate them to the air above. Theoretically, we would expect nothing pernicious to be evolved from the sea, the surface of which is always in a state of greater or lesser agitation; and practically we find the conclusion to be just. On shore, on the contrary, we have all varieties of soil, in many conditions of which we have a right to infer, that gaseous evolutions will take place by the action of heat, and experience but too plainly tells us, that wherever certain conditions are present within the tropics, there, in general, disease is most rife. It will no doubt be said that we have, as yet, no chemical evidence of the existence of malaria. But because its precise nature is unknown to us, are we, in the face of such destructive results, to deny its being? We may just as well say, that small-pox and other exanthemata cannot be propagated through the medium of the atmosphere, although the constitution of their poisons has not as yet been recognised by any 'chemist or physiologist.'

"Provided that men have not been for a considerable time exposed to the noxious exhalations within rivers, it seems abundantly evident that their effects are in a great measure counteracted by the air of the open sea.

"In November, 1838, H.M.S. *Pylades* (a ship remarkable for her general salubrity), under the command of Captain William L. Castle, had occasion to be in the Bonny about forty-eight hours; several of her crew were attacked with fever, soon after leaving the river, but they speedily recovered on the passage to Saint Helena, to which island the ship was ordered. Capt. Castle has observed similar results in other ships during a long period of service on the west coast.

"In March 1839, Capt. Craigie was again called into the Bonny, to settle some disputes between King Peppel and the palm-oil ships. Dreading a repetition of the calamities of 1837, he left the *Scout* outside, and proceeded up the river in the *Bonetta*, brigantine, commanded by Lieutenant (now Commander) John Stoll, taking with him, in addition to her crew, Lieut. Frederick A. Campbell, Mr. Bainbridge, mate, and myself. We remained off the town about thirty-six hours. The day on which we got out of the river, upwards of twelve of the seamen and marines presented themselves to Mr. Kinnear, the medical officer of the *Bonetta*, complaining of heat of skin, vomiting, and other febrile symptoms, which, however, soon disappeared as the vessel proceeded to sea.

"From these and numerous other instances, it would appear that the action of miasma is quite analogous to that of other poisons, inasmuch as its injurious effect is in proportion to the amount taken into the system. By remaining long in rivers, the quantity imbibed will be very commonly sufficient to destroy life, while a short stay in such localities will only produce a temporary disorder of the functions."

